# Assessment of current colostrum feeding practices and passive transfer of immunity in dairy herds in Great Britain: A cross‐sectional study

**DOI:** 10.1002/vetr.6025

**Published:** 2025-11-26

**Authors:** George Lindley, Richard E. Booth, D. Claire Wathes, Nicola Blackie

**Affiliations:** ^1^ Department of Pathobiology and Population Sciences Royal Veterinary College Hatfield Hertfordshire UK

**Keywords:** calf, dairy, immunity, passive transfer

## Abstract

**Background:**

Updated targets for measuring transfer of passive immunity (TPI) at the herd level have been suggested, but the current performance of dairy herds in Great Britain is unknown.

**Methods:**

A cross‐sectional study was performed. Serum total protein (STP) data collected between October 2022 and October 2023 by 21 veterinary practices were benchmarked. Questionnaires were also distributed to collect information about colostrum feeding, calf husbandry and veterinary engagement, and associations between these factors and herd TPI were analysed using ordinal logistic regression.

**Results:**

A total of 4455 STP results from 91 herds were collected. Overall, 26% of calves had an STP concentration of less than 52 g/L (*n* = 1144). The number of herds with less than 10% of measured calves having an STP concentration of less than 51 g/L was 26% (*n* = 24). In contrast, 44% of herds (*n* = 40) recorded more than 40% calves having an STP concentration of 62 g/L or more. Herd TPI score was negatively associated with colostrum replacer feeding (odds ratio 0.09, 95% confidence interval: 0.01–0.54, *p* = 0.01).

**Limitations:**

A limited number of veterinarians and farmers completed the accompanying questionnaire, increasing the likelihood of Type I statistical error. Survey responses may be influenced by response bias.

**Conclusion:**

The overall herd TPI measured in the study was inadequate, suggesting that renewed emphasis on the optimisation of TPI within British dairy herds is necessary.

## INTRODUCTION

Transfer of passive immunity (TPI) describes the process of colostrum ingestion and enteric absorption of immune components, including immunoglobulin (Ig), by the neonate. During gestation, the synepitheliochorial structure of the bovine placenta ensures isolation of fetal and maternal components, inhibiting the transfer of macromolecules, including Ig, in utero.^1^, ^2^ The newborn calf is therefore born agammaglobulinaemic and relies on the passive absorption of Ig in colostrum as a source of maternally derived antibody while endogenous production begins to increase during the preweaning period.^3^, ^4^ Colostrum management is the most important factor influencing calf health and survival at this stage and may possibly be associated with longer‐term benefits to growth and adult productivity.^5^ When an insufficient mass of colostral Ig is absorbed (<150–200 g), the serum Ig titres remain low, increasing calves' susceptibility to pneumonia, diarrhoea, colisepticaemia and death.^5^, ^6^, ^7^, ^8^, ^9^

In order to minimise this risk, calf‐level cut‐offs to define ‘failure’ of TPI, as well as herd‐level thresholds, using multiple diagnostic modalities have been suggested.^10^, ^11^ For example, a serum total protein (STP) threshold of 52 g/L was suggested as a dichotomous cut‐off, as lower values were attributed to a greater risk of colisepticaemia.^6^, ^12^ STP concentrations may also be compared with results from a representative subset of calves to assess whether failure of TPI is prevalent at the herd level.^11^, ^13^, ^14^, ^15^, ^16^ While radial immunodiffusion (measuring serum IgG) is considered the reference standard for assessing TPI, the STP concentration is a commonly used indirect measure of passive immunity due to its low cost, quick turnaround time and the ability to perform testing on‐farm. However, the association between TPI, disease and performance is not binary, and higher titres of serum IgG or STP are associated with reductions in morbidity.^6^, ^17^, ^18^, ^19^ On this basis, North American consensus proposed a revised classification system for TPI, whereby calves were categorised as ‘poor’, ‘fair’, ‘good’ or ‘excellent’ based upon a serum IgG concentration of less than 10.0 g/L, 10.0–17.9 g/L, 18.0–24.9 g/L and 25 g/L or more, respectively. These are equivalent to STP concentrations of less than 51 g/L, 51–57 g/L, 58–61 g/L and 62 g/L or more, respectively.^20^ Using this updated grading system, an ordinal relationship between TPI category at the calf‐level and morbidity has been demonstrated in several recent studies, with higher titre categories associated with improved health and growth outcomes.^21^, ^22^, ^23^

Alongside individual‐calf TPI targets, Lombard et al.^20^ also suggested herd‐level targets for the percentage of calves within each category. These targets were based upon data collected during 2014 by the National Animal Health Monitoring System (NAHMS), a stratified, random, US‐based survey that provides weighted results reflective of the national population.^24^ They suggest that the herd‐level percentage of calves within the poor, fair, good and excellent categories should be less than 10%, ~20%, ~30% and greater than 40%, respectively. While calf‐level passive transfer data from dairy herds located within Great Britain (GB) have been reported, predominantly in the form of STP concentrations, no equivalent measurement of herd‐level TPI performance has been calculated, and therefore, the comparability of the aforementioned targets to GB herds is unknown.^25^, ^26^, ^27^, ^28^ Therefore, the main objective of this study was to measure TPI in calves born in GB dairy herds using STP and to compare their performance with the recent guidelines described by Lombard et al.^20^ A secondary objective was to describe and characterise calf and colostrum management practices in GB dairy herds and test their association with herd TPI (HTPI) performance.

## MATERIALS AND METHODS

This study was approved by the Social Sciences Research Ethical Review Board (URN SR2022‐0124) of the Royal Veterinary College, London, UK.

### Questionnaire design

A total of 37 questions regarding herd details (e.g., size, calving pattern and yield, *n* = 10 questions), labour (e.g., number of calf rearers, *n* = 3 questions), calving and colostrum feeding (e.g., feeding methods and timings, *n* = 18 questions), and veterinary involvement (e.g., frequency of visits, interest in calf health and current TPI monitoring, n = 6 questions) were drafted into a questionnaire (Supporting Information S1). This draft was reviewed by a group of veterinarians and scientists (*n* = 4) with expertise in dairy farming, and amendments were made thereafter to produce a final version. Not all participants responded to every question. Accordingly, response frequencies were reported for each question, with percentages calculated based on the number of respondents to that specific item. Response frequencies are also described in Supporting Information S1.

### Practice and farm recruitment

A cross‐sectional study was performed, using data collected as routine practice by veterinarians between October 2022 and October 2023. The population of interest was British veterinary practices with dairy farmer clients routinely collecting STP data. The package ‘Hmisc’ in R was used to estimate the required number of enrolled herds with accompanying questionnaire responses.^29^ This function estimates the required sample size for comparing ordinal outcomes under the proportional odds logistic regression model. In our study, the outcome of interest was the HTPI score, an ordinal variable with four categories (Poor, Fair, Good, Excellent). The calculation used the proportions suggested by Lombard et al.^20^ as the best available estimate of the marginal probabilities (Poor = 0.1, Fair = 0.2, Good = 0.3, Excellent = 0.4). The desired power was 0.8 and *α* = 0.05. An odds ratio value of 2.5 or 0.4 was estimated to represent a clinically meaningful effect size. Based on this calculation, 32 herds were required. Veterinarians and practices located throughout GB were contacted directly by the primary researcher (G.L.). In addition, interested individuals were encouraged to contact the researchers following advertisements within published literature^30^, ^31^ and a social media campaign (using LinkedIn, X [formerly Twitter], Instagram and Facebook). The questionnaire and farmer consent forms were shared with participating practices, and veterinarians were requested to discuss the project with dairy clients who routinely monitored their herd STP data. Consent to share STP data was collected by veterinarians, who also completed a questionnaire with each enrolled herd. At the end of the study period, veterinarians were contacted, and all available data were collated by the researchers.

An additional subset of STP data was collected from practice records without accompanying questionnaire data. In this case, consent was sought from practices and personal data were anonymised before data sharing, but a herd identifier was assigned to each animal. Herds without corresponding questionnaire data were not included in the inferential statistical analyses. Data were obtained initially from 33 herds, and data from a further 58 herds were collected from practice records.

### Inclusion criteria

All STP data collected from calves between 1 and 7 days of age born of dairy cow origin were eligible for inclusion. This included male and female dairy calves and dairy x beef calves. Where possible, additional data, including breed and sex, were also recorded. For data collected from veterinary records, descriptive information was anonymised, and records were only included if they could be attributed to a specific herd, from calves born in dairy herds and aged between 1 and 7 days. To minimise erroneous conclusions, herds were only included in the analysis if a minimum of six STP samples were available. While the cut‐off of six was selected arbitrarily, it was intended to ensure a more stable and meaningful estimate of herd‐level performance.

### Data analysis

Data were collated using Excel (Microsoft version 16.77.1, 2023), and descriptive statistics were produced. Initially, STP concentration was considered as a continuous variable and visualised by plotting a histogram. Thereafter, it was transformed into a categorical variable subdivided into four groups, as described by Lombard et al.^20^: ‘poor’ (<51 g/L), ‘fair’ (51–57 g/L), ‘good’ (58–61 g/L) and ‘excellent’ (≥62 g/L). To evaluate the herd‐level performance, the proportion of calves on each farm classified within each TPI category was determined. Following which, a modified version of the guidelines proposed by Lombard et al.^20^ was used with data benchmarked overall and by herd to assess the herds' ability to achieve the HTPI targets of less than 10% of calves with an STP concentration of less than 51 g/L, less than 20% of calves with an STP concentration between 51 and 57 g/L, more than 30% of calves with an STP concentration between 58 and 61 g/L, and greater than 40% of calves with an STP concentration of 62 g/L or more. The analysis of quantitative data was performed in R.

All available questionnaire responses were collected and written into Excel (Microsoft version 16.77.1, 2023). For time‐based questions receiving multiple responses (e.g., Q: ‘How long after birth is colostrum harvested from the cow? (hours after birth)’, A: 1–6 hours), minimum and maximum responses were recorded. Free‐text responses were categorised (e.g., Q: ‘What do you do if the quality of colostrum is not sufficient?’, A: ‘Use frozen’, ‘Supplement with colostrum replacer (CR)’, ‘Feed with CR only’, ‘Increase volume’, ‘Feed anyway’).

For inferential analysis, a one‐point score was assigned for each suggested HTPI target and each individual herd to produce a total herd score of between 0 and 4. For example, if a herd achieved less than 10% of calves with an STP concentration of less than 51 g/L but did not fulfil the other HTPI targets, it would be scored ‘1’. An ordinal logistic regression model was fitted using the ‘MASS’ package in R, to estimate associations between the outcome variable of herd score and responses provided in the accompanying questionnaire.^32^

Candidate explanatory variables were initially considered univariately and compared to a null (intercept only) model using a chi‐squared test (Supporting Information S2; Table S1). Obtaining a test *p*‐value of less than 0.25 was used as a threshold for adding variables into a multivariable model (Supporting Information S2; Table S2). Subsequently, the final model was determined by applying the forward stepwise selection procedure, with variables being included based upon improvement in the Akaike information criterion (AIC). The significance of the final model was assessed by comparing its deviance with that of the null model through a likelihood ratio test.

Variance explained by the model was determined by the calculation of pseudo‐*R* ^2^ values using the DescTools package in R. The proportional odds assumption was checked with Brant's test, using the R package brant.^33^, ^34^ Finally, goodness of fit was assessed using the Hosmer–Lemeshow test included in the R package generalhoslem.^35^

## RESULTS

### Practice and farm characteristics

**FIGURE 1:**
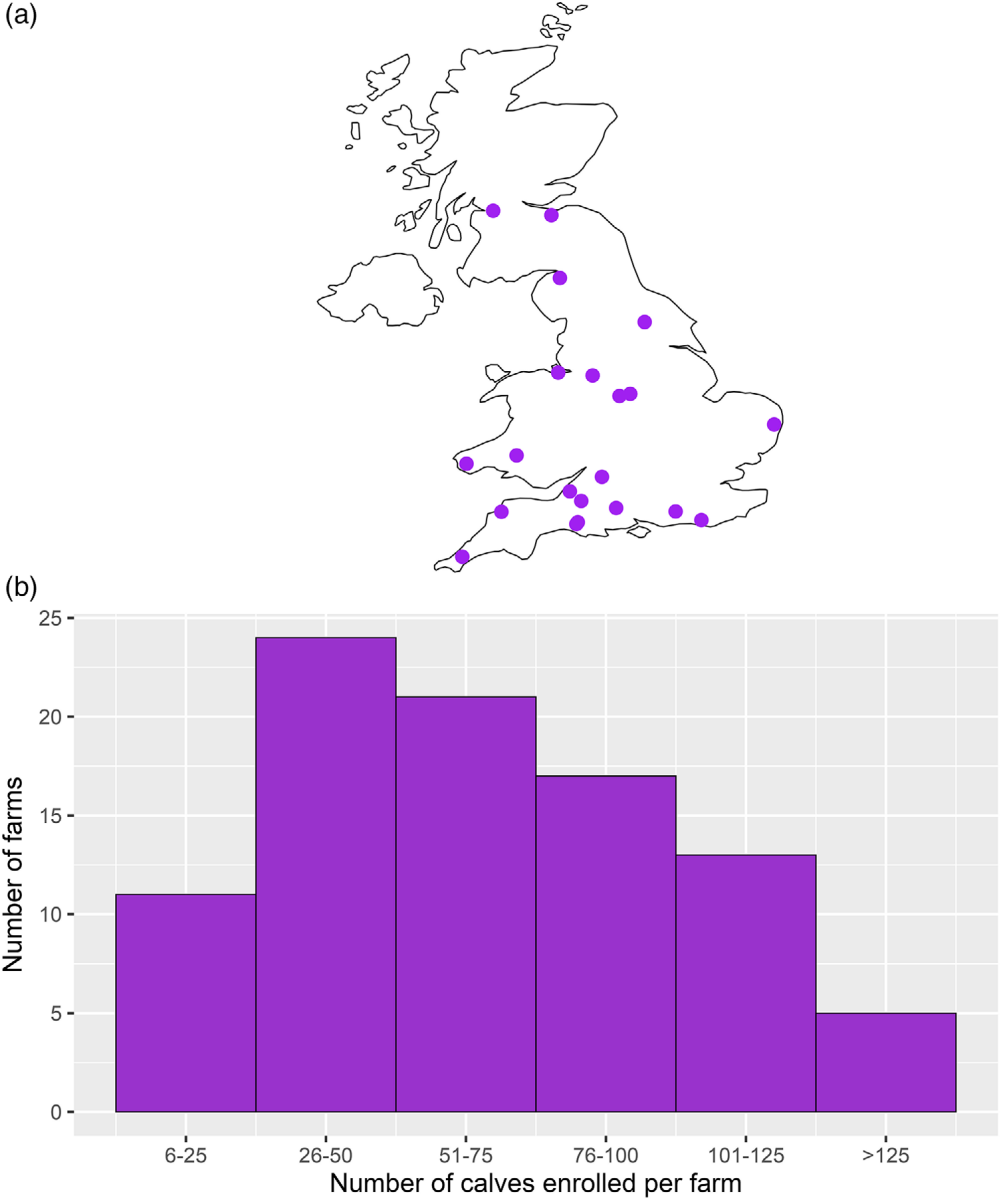
(a) Map showing the distribution of enrolled veterinary practices (*n* = 21) within Great Britain. (b) Histogram depicting the distribution of the number of calves enrolled per farm from the 91 farms participating in the study.

A total of 21 veterinary practices located throughout GB were recruited (Figure 1a), generating data from 111 farms. After exclusion of herds with less than six STP samples, a total of 91 farms remained available for analysis. The median number of farms enrolled per practice was two (interquartile range [IQR] 1–4), and the median number of calves enrolled per farm was 24 (IQR 15–40) (Figure 1b). Calves enrolled from herds with questionnaire data accounted for 53% (*n* = 2413) of the total number (*n* = 4555). Breed and sex data were available for 47% (*n* = 2084) and 61% (*n* = 2728) of calves, respectively. Female calves predominated (70%, *n* = 1909). Holstein Friesian was the most common dairy breed, accounting for 61% (*n* = 1242) of calves, whereas for the beef cross calves, Aberdeen Angus sires were the most common (13%, *n* = 281).

### Questionnaire responses

Data from 33 dairy herds across 16 veterinary practices, collected by 21 veterinarians, representing 51% of all enrolled calves (*n* = 2413), were available for analysis. Respondents were predominantly from conventional (non‐organic), year‐round calving, dairy‐only farms with Holstein or Holstein Friesian herds (Table 1). The median herd size was larger than average in GB (median 264 milking cows, range: 104–2500), and the median 305‐day milk yield was 10,452 L (range: 5500–13,810 L).^36^ In the study population, 44% of farms (12/27) employed at least one calf rearer per 100 annual calvings (≥1:100), while the remaining 56% (15/27) had fewer than one calf rearer per 100 calvings (<1:100). Most calf rearers were aged 31–40 (35%, *n* = 12/35) and performed other duties additional to youngstock care (64%, *n* = 21/32). Over half of herds (64%, *n* = 21/33) vaccinated pregnant cows, with the main indication cited as passive immunisation of calves against neonatal diarrhoea (81%, *n* = 17/21).

**TABLE 1:** Demographics of farms (*n* = 33) with accompanying questionnaire data.

Variable	Frequency	Percentage^a^
**Farm type**		
Dairy only	30	91
Dairy and other^b^	3	9
No answer	0	
**Enterprise type**		
Conventional	31	97
Organic	1	3
No answer	1	
**Calving pattern**		
All year round	24	73
Autumn block	4	12
Spring block	2	6
Autumn and spring	3	9
No answer	0	
**Predominant breed**		
Holstein Friesian	12	41
Pedigree Holstein	13	45
Other crossbreed	4	14
No answer	4	
**Under movement restrictions** ^c^		
Yes	9	27
No	24	73
No answer	0	
**Vaccination of pregnant cows**		
Yes	21	64
No	12	36
No answer	0	

^a^ Percentage values were calculated from available responses to each question.

^b^ Other **=** non‐bovine operations.

^c^ Statutory movement restrictions associated with *Mycobacterium bovis* (bTB) test status may be implemented under UK legislation.

Cows commonly calved within a dedicated area (77%, *n* = 23/30) in groups (65%, *n* = 20/31). After birth, most farms kept calves with their dam for up to 24 hours (94%, *n* = 30/32) and harvested colostrum within 6 hours (94%, *n* = 16/17). The predominant method of feeding colostrum was almost evenly split between bottle, oesophageal tube and suckling from the dam (Table 2). A first colostrum feed of between 3.5 and 4.5 L was common (58%, *n* = 14/25), as was the provision of a second feed (58%, *n* = 18/31). Colostrum quality was usually tested, with the Brix refractometer reportedly used most frequently (86%, *n* = 25/29), and a Brix threshold of 22% most commonly used as a minimum quality cut‐off (range: 15–25%). Various actions were taken when colostrum quality was insufficient (Table 2).

**TABLE 2:** Details regarding colostrum feeding and transfer of passive immunity for the 33 dairy herds with accompanying questionnaire data.

Variable	Frequency	Percentage^a^
**Predominant colostrum feeding method**		
Left to suckle	9	27
Bottle	12	39
Oesophageal tube	11	33
No answer	1	
**Is the quality of colostrum tested?**		
Yes	23	70
Sometimes	5	15
No	5	15
No answer	0	
**How is colostrum quality tested?**		
Brix	25	87
Hydrometer	2	7
Colostroballs	1	3
Visually	1	3
No answer	4	
**What do you do if the quality of colostrum is not sufficient?**		
Mix with colostrum replacer	7	27
Feed colostrum replacer (only)	5	19
Feed stored colostrum or available colostrum from an alternative cow (including pooled)	10	38
Increase the volume fed	3	12
Feed anyway	1	4
No answer	7	
**Before this study, have blood samples ever been taken from calves to determine the extent of passive transfer of immunity?**		
Yes	31	94
No	2	6
No answer	0	
**If yes, how frequently are samples collected on your farm?**		
More than every 3 months	20	64
Every 3–6 months	3	10
Every 6–12 months	3	10
Less than once a year	5	16
No answer	2	
Proportion of calves sampled: Number of annual calvings		
1 calf per 1–10 annual calvings	21	73
1 calf per 11–20 annual calvings	4	14
1 calf per 21–30 annual calvings	0	0
1 calf per 31–40 annual calvings	3	10
1 calf per 41–50 annual calvings	1	3
No answer	4	

^a^ Percentage values were calculated from available responses to each question.

The majority of enrolled herds took blood samples to assess for TPI more frequently than every 3 months (72%, *n* = 22/31). No significant association between TPI blood sampling frequency and annual number of calvings was found (Spearman's correlation coefficient: rho = −0.2, *p* = 0.29). Although data on the respondents' calf sampling strategy were unavailable, information on the proportion of calves sampled relative to the total number of annual calvings was measured, with the majority of herds measuring TPI in at least one calf every 1–10 calvings (Table 2). Advice regarding calf health was frequently provided by attending veterinarians, most frequently on a weekly basis (32%, *n* = 10/31) or between a fortnightly and monthly basis (39%, *n* = 12/31). All respondents found their veterinarians to be interested in calf health, although a minority desired greater support from their veterinarian (23%, *n* = 7/31).

#### Transfer of passive immunity

**FIGURE 2:**
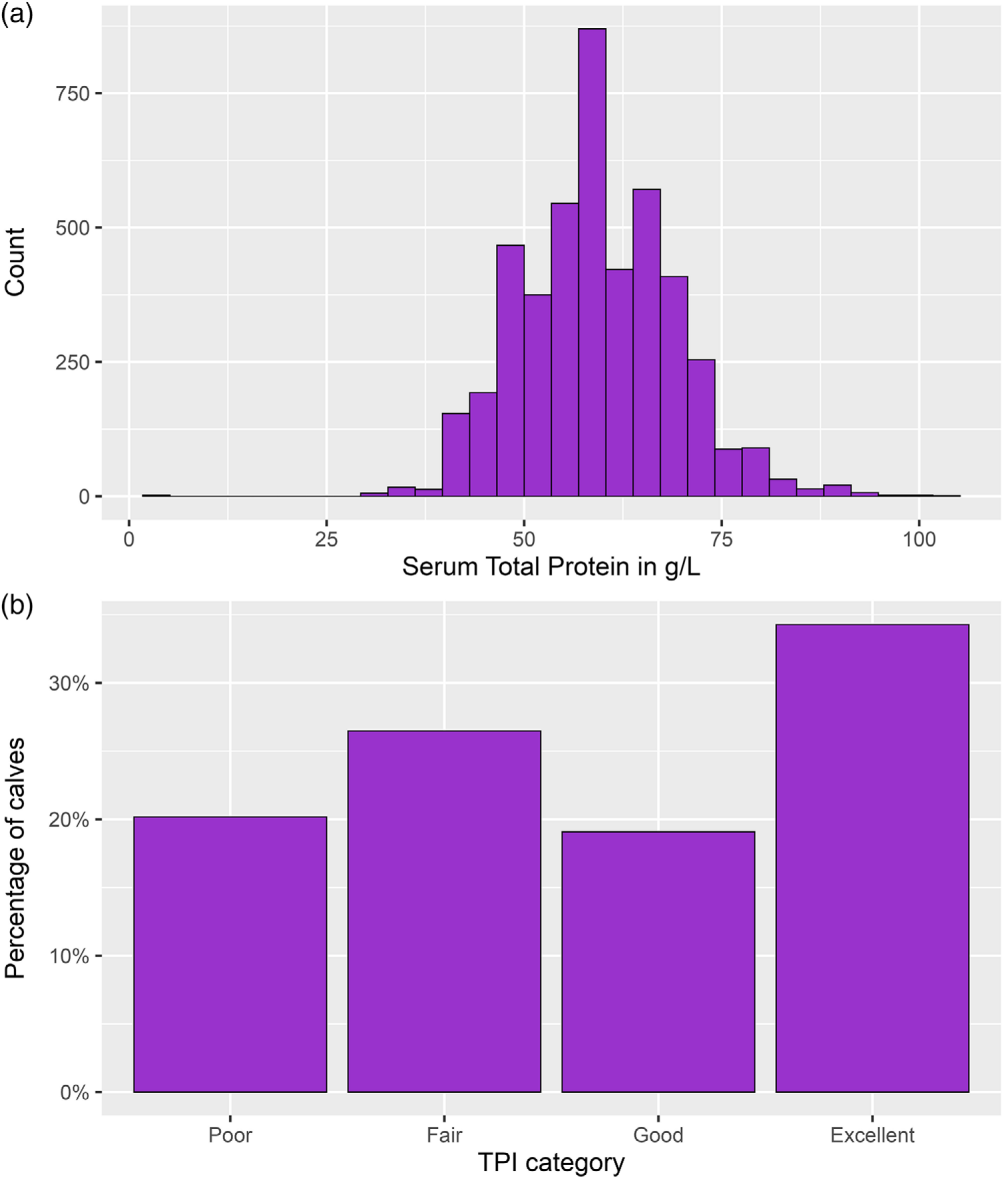
(a) Histogram of the serum total protein concentrations of all enrolled calves (*n* = 4452). (b) Overall percentages of calves classified as having ‘poor’ (<10% <51 g/L), ‘fair’ (<20% 51–57 g/L), ‘good’ (>30% 58–61 g/L) and ‘excellent’ (>40% ≥62 g/L) transfer of passive immunity (TPI), as described by Lombard et al.^20^

**FIGURE 3:**
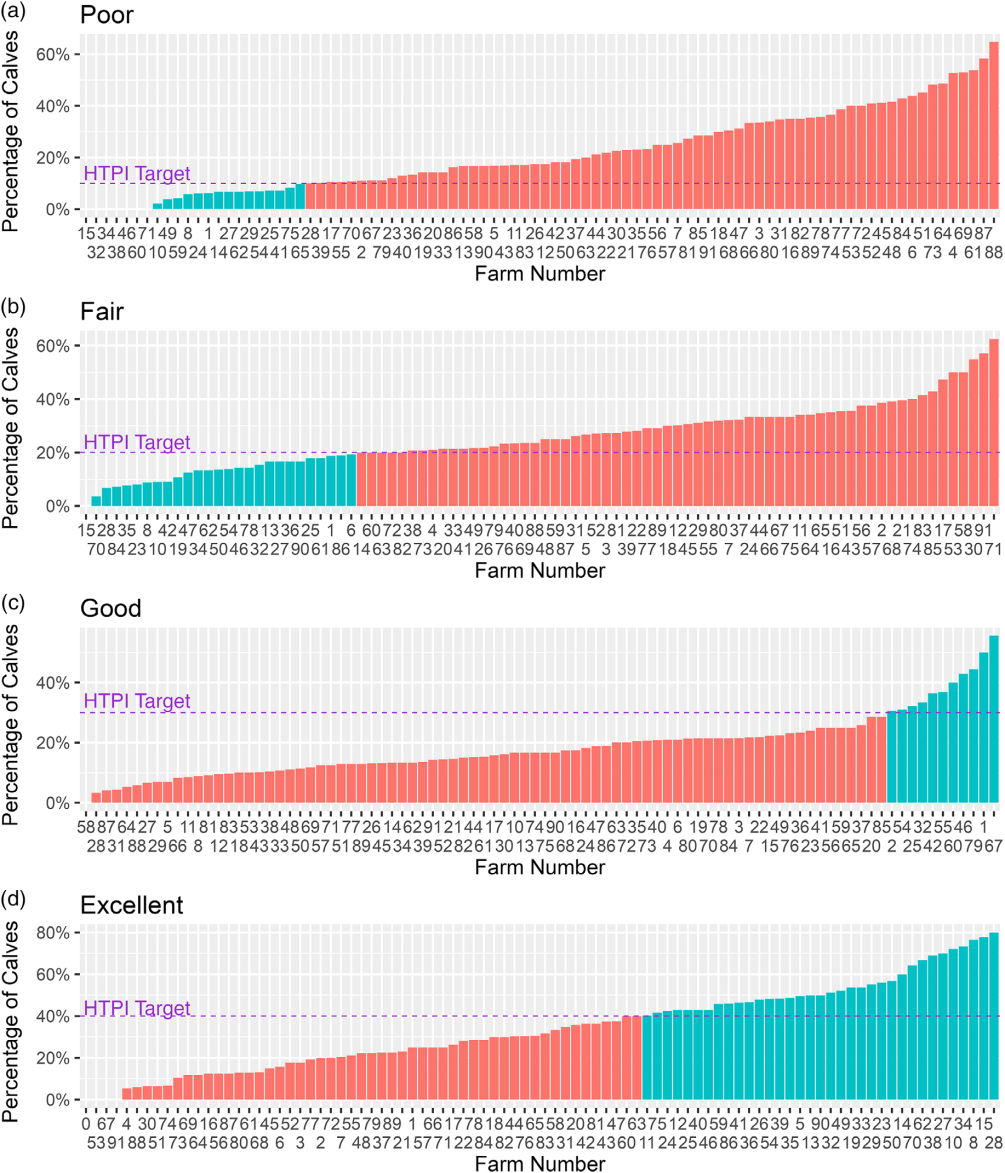
Herd‐level transfer of passive immunity (HTPI) performance, measured using serum total protein concentrations collected and reported by collaborating veterinary practices (*n* = 21), described as the proportion of calves (*n* = 4452) within each herd (*n* = 91) meeting the ‘poor’ (<10% <51 g/L), ‘fair’ (<20% 51–57 g/L), ‘good’ (>30% 58–61 g/L) and ‘excellent’ (>40% ≥62 g/L) guidelines, as described by Lombard et al.^20^ Each herd is depicted as a bar, and the suggested transfer of passive immunity target is shown as a horizontal purple line. Herds in red did not meet the suggested target, whereas those in blue did meet the guidelines.

**FIGURE 4:**
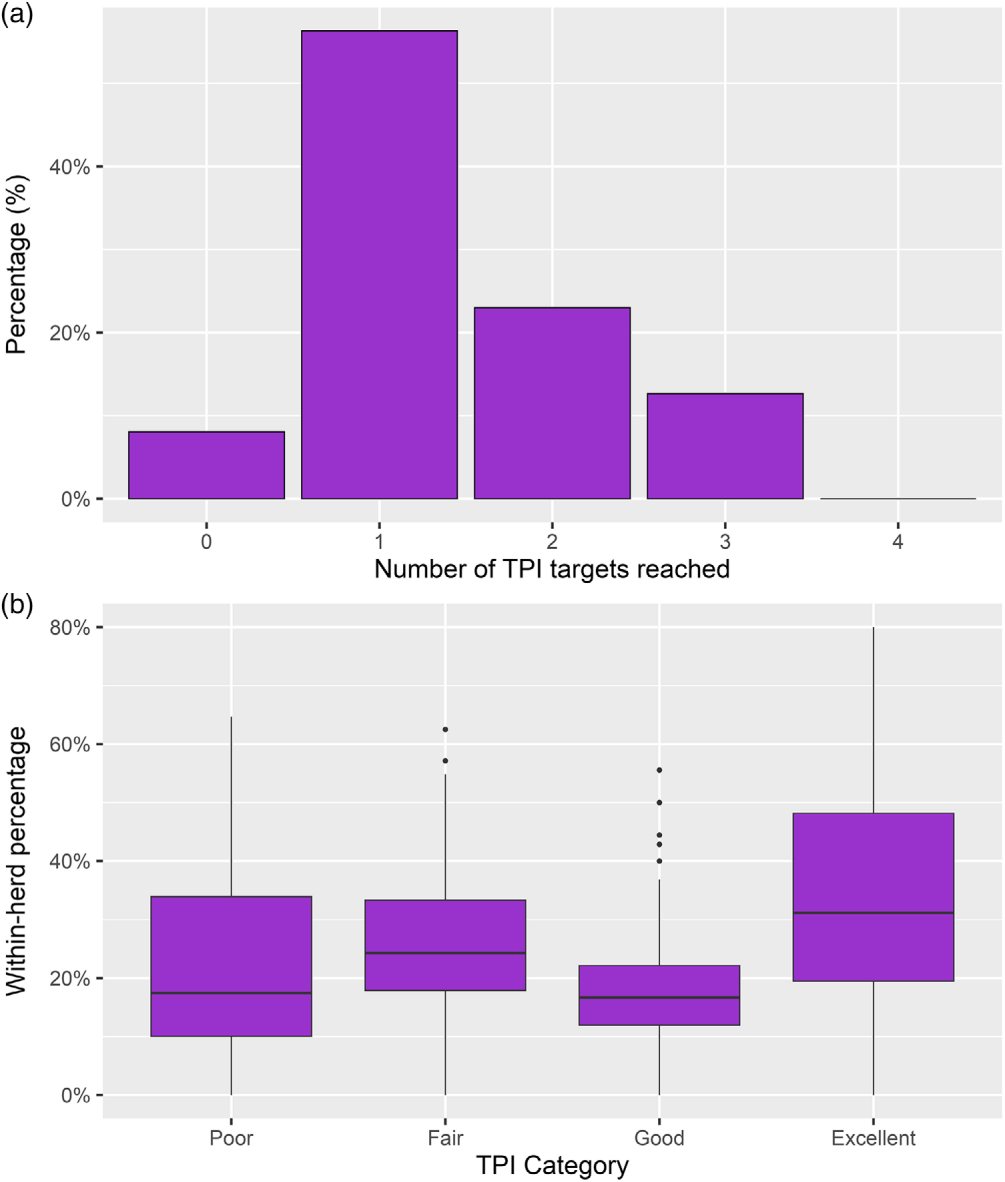
(a) Bar graph showing the percentage of herd‐level transfer of passive immunity (TPI) targets achieved by 91 herds located throughout Great Britain. Herds were assigned a one‐point score for each of the TPI targets they achieved (0–4). Targets were described, in terms of serum total protein concentration, as <10% <51 g/L (‘poor’), <20% 51–57 g/L (‘fair’), >30% 58–61 g/L (‘good’) and >40% ≥62 g/L (‘excellent’), as described by Lombard et al.^20^ (b) Boxplot showing the within‐herd percentage of calves within each TPI category. The horizontal line within each box marks the median value, the lower and upper edges indicate the first and third quartiles, and the whiskers are 1.5 times the interquartile range. Points outside of this distribution are shown as dots. Herds with no calves within a specific category were omitted from the analysis.

Based upon a cut‐off of 52 g/L, 26% of calves within the study were deemed to have ‘failure’ of passive transfer (*n* = 1144/4452) (Figure 2a). Overall, once categorised as described by Lombard et al.,^20^ 20% of calves were classified as ‘poor’ (*n* = 917/4452), 26% as ‘fair’ (*n* = 1206/4452), 19% as ‘good’ (*n* = 869/4452) and 34% as ‘excellent’ (*n* = 1560/4452) (Figure 2b). When the individual‐herd performance was assessed for each TPI guideline, 26% (*n* = 24/91), 35% (*n* = 32/91), 14% (*n* = 13/91) and 44% (*n* = 40/91) met the ‘poor’, ‘fair’, ‘good’ and ‘excellent’ targets, respectively (Table 3 and Figure 3). After herds were assigned a one‐point score for each TPI target they achieved, the majority of herds (54%, *n* = 50/91) achieved a single point. A decreasing number of herds achieved two points (23%, *n* = 21/91), three points (13%, *n* = 12/91), or zero points (9%, *n* = 8/91). No herd achieved four points (Figure 4).

**TABLE 3:** Summary of herd‐level transfer of passive immunity (TPI) performance for all 4452 calves sampled from the 91 enrolled herds.

TPI category	Target STP (g/L)	Herds meeting the target		Herds not meeting the target		Median herd percentage (IQR)
		Number	Percentage	Number	Percentage	
Poor	<10% <51	24	26	67	74	17 (24)
Fair	<20% 51–57	32	35	59	65	24 (15)
Good	>30% 58–61	13	14	78	86	17 (10)
Excellent	>40% ≥62	40	44	51	56	32 (28)

Abbreviations: IQR, interquartile range; STP, serum total protein.

#### Inferential analysis

Based on the 33 recruited herds for which questionnaire results were available, the final model included a single predictor. Herds that fed colostrum replacer (*n* = 12) were significantly less likely to achieve higher HTPI scores than those that did not (odds ratio 0.09, 95% confidence interval: 0.01–0.54, *p* = 0.01). Based on the threshold coefficients, this effect was significant for scores between 0 and 2, but not for higher scores (Table 4). Together, the predictors accounted for a significant amount of the variance in herd score, as evidenced by a *χ* ^2^ likelihood ratio value of 7.81 (*p* = 0.005). Overall, the model accounted for between 23% and 24% of the variance in the outcome, as determined by Cox–Snell and Nagelkerke pseudo‐*R* ^2^ values of 0.23 and 0.24, respectively. The proportional odds assumption was not breached, and model fit was good (Hosmer–Lemeshow *χ* ^2^ = 43.5, *p* = 0.15).

**TABLE 4:** Results of the final ordinal logistic regression model, showing covariates associated with the outcome measure of herd‐level transfer of passive immunity (TPI) performance when herds were ranked according to the number of TPI targets (0–4) that they met, defined as <10% <51 g/L (‘Poor’), <20% 51–57 g/L (‘Fair’), >30% 58–61 g/L (‘Good’), 40% ≥62 g/L (‘Excellent’), as modified from Lombard et al.^20^

Variable	Response	Value (95% CI)	*p‐*value	OR (95% CI)
Do you feed colostrum replacer?	Yes	−2.14 (−4.21 to 0.62)	0.01	0.09 (0.01–0.54)
0|1		−2.96	0.00	
1|2		−1.96	0.02	
2|3		−0.17	0.82	
3|4		1.26	0.15	

*Note*: The rows labelled 0|1, 1|2, 2|3, 3|4 are the intercepts (or cutpoints) in the ordinal logistic regression model. They represent the boundaries between the two ordered outcome categories displayed in each row (i.e. 0|1, herd transfer of passive immunity (HTPI) score 0 vs. scores ≥1; 1|2, HTPI score ≤1 vs. scores ≥2; 2|3, HTPI score ≤2 vs. scores ≥3 and 3|4, HTPI score ≤3 vs. score 4).

Abbreviations: CI, confidence interval; OR, odds ratio.

## DISCUSSION

This study measured and benchmarked the herd‐level TPI performance of 91 British dairy herds between October 2022 and October 2023 in order to compare their performance with the recent guidelines described by Lombard et al.^20^ The modal HTPI score was just one point, which was achieved by 54% of herds (*n* = 50/91), showing that only a minority of herds was able to achieve the passive transfer standards suggested by Lombard et al.^20^ Herds most commonly fulfilled the ‘excellent’ benchmark of greater than 40% of calves with an STP concentration of 62 g/L or more (44%, *n* = 40/91). The ‘fair’ benchmark of less than 20% of calves with an STP concentration between 51 and 57 g/L was the second most‐achieved goal (35%, *n* = 32/91), suggesting that a bimodal distribution of HTPI exists. This study demonstrated that while herds can achieve ‘excellent’ HTPI, renewed focus on reducing the percentage of ‘poor’ and ‘fair’ calves is needed. These guidelines were proposed to be reflective of dairy calf rearing within the United States of America, using data from the NAHMS 2014 calf category survey to ensure their achievability, as evidenced by the fact that between 2014 and 2015, 32% (*n* = 33/103) of sampled herds achieved all of the standards.^37^ At the individual level, Sutter et al.^21^ found calves with excellent TPI (measured using Brix refractometry) had a reduced likelihood of developing pneumonia or morbidity compared with those with poor TPI, while another study found a decreasing hazard of diarrhoea with improving TPI category, and a significantly greater risk of pneumonia and death in calves with poor TPI compared to those with excellent TPI (*p* = 0.02 and *p* < 0.001, respectively).^38^ On this basis, improving HTPI status is a crucial means of reducing calfhood morbidity and mortality, and thereby achieving improved growth outcomes and productivity.^39^

The present study enrolled a similar number of herds to the NAHMS 2014 calf category survey, albeit as a convenience sample of willing respondents, and found that no herd was able to achieve all four TPI targets (Figure 4). Although the present study benchmarked HTPI using the thresholds proposed by Lombard et al.,^20^ alternative HTPI thresholds have been published. McGuirk^13^ suggested that failed TPI at the herd level should be diagnosed if more than 20% of sampled calves had serum IgG concentrations of less than 10 g/L (equivalent to the ‘poor’ category of <51 g/L), whereas the NAHMS 2014 report originally presented a simplified threshold requiring greater than 90% of calves to achieve an STP concentration of 52 g/L or greater.^37^ In the present study, 74% (*n* = 67) of herds would fail based upon the former threshold, while only 25% (*n* = 23) of herds passed the latter threshold. Nonetheless, the results of this study are similar to other findings and suggest that the categories described by Lombard et al.^20^ are not only relevant to North American systems. Recent evaluation of the HTPI performance of farms within six European countries found that only one in 108 achieved the ‘excellent’ herd target, compared to one in 91 in this study.^40^ In contrast, a study of a single large German herd described a greater median percentage of calves within the ‘excellent’ category and a lower number of calves within the ‘poor’ category in comparison to this study (Table 3).^21^ Similar categorisation has also been described in Spain and Australia.^41^, ^42^

Although many studies have investigated factors associated with TPI status in the individual calf, fewer data are available investigating HTPI associations. A pan‐European study associated HTPI with the volume and quality of colostrum delivered on‐farm, although, notably, no discrimination between maternal or replacement sources was described.^40^ The present study combined herd‐level TPI measures with questionnaire responses to investigate factors associated with TPI performance. We found that herds that fed colostrum replacer had a significantly reduced likelihood of achieving a higher HTPI score. This outcome is similar to that of a Japanese study, which investigated 39 herds and found that herd‐level failure of passive transfer was significantly associated with the use of colostrum replacer instead of frozen colostrum.^43^ Unfortunately, individual calves fed colostrum from maternal or replacement sources could not be differentiated in the present study, as these data were not reported. This would have been useful when extrapolating serum IgG concentration from STP values, especially as differences in their composition may affect the indirect measurement of IgG concentration.^43^, ^44^

This study aggregated STP data collected and reported by veterinary practitioners. One challenge with the interpretation of these data is that calves were selected for sampling at the discretion of cooperating veterinarians. However, as calf age was available for most calves, it was possible to include only animals between 1 and 7 days old. Refractometry is also liable to misclassification errors as a consequence of dehydration or ongoing inflammatory processes, which result in false elevations in the results and could lead to an overestimation of the herd‐level TPI performance.^45^, ^46^ On the other hand, indirect methods of TPI measurement have been suggested to underestimate IgG concentrations. Furthermore, the accuracy of refractometry is reliant on effective calibration and interpretation, which cannot be verified. Ideally, STP measurement should be conducted only in healthy calves and can be accompanied by simultaneous assessment of hydration and inflammatory parameters.^47^, ^48^ The results of the present study should be interpreted considering these possible causes of inaccuracy.^49^ Furthermore, this study enrolled herds that were routinely collecting STP data, and there may be an unknown level of population bias. This could happen if these herds represented either a proactive subset engaged in continual improvement of HTPI or a subset with a history of poor TPI performance that required long‐term follow‐up. This may partially explain the wide variation in the within‐herd proportions (Table 3), although similar variability in STP concentrations has previously been demonstrated in British herds.^28^

The inferential statistics performed in this study were limited by the distribution of herds achieving TPI categories, as herds modally achieved a score of one with an uneven distribution in other score categories (Figure 4). This increased the risk of overparameterisation in this study, which we minimised by using a forward stepwise design.

To avoid the risk that STP data were not representative of the true within‐herd proportion of calves meeting the HTPI categories, herds with less than six available STP samples were excluded, although this number was selected arbitrarily. Power analyses were performed a priori in this study, and the number of questionnaire responses received satisfied the calculation. Nonetheless, there is a risk of Type II statistical error having occurred, and future studies should ensure sufficient sample size to account for the distribution of HTPI scores found herein. An additional limitation of this study is that survey responses may be influenced by response bias.

## CONCLUSIONS

The results of this study, which aggregated STP data from 4552 calves across 91 herds, suggested that the HTPI performance of British dairy herds does not meet previously suggested benchmarks. Ordinal logistic regression identified a single predictor associated with the outcome measure of HTPI score, with herds feeding colostrum replacer significantly less likely to achieve higher HTPI scores. A renewed emphasis on HTPI may be necessary for improvements to be achieved.

## AUTHOR CONTRIBUTIONS

George Lindley, Nicola Blackie, Richard Booth and Claire Wathes contributed to the conceptualisation of the study. George Lindley collected and analysed the data and drafted the original manuscript. Nicola Blackie, Richard Booth and Claire Wathes edited the manuscript. All authors have read and approved the final manuscript.

## CONFLICT OF INTEREST STATEMENT

George Lindley has previously been a paid speaker by Krka, who had no role in the study design, interpretation or decision to submit this manuscript for publication. The remaining authors declare that they have no conflicts of interest.

## ETHICS STATEMENT

This study was prospectively approved by the Social Sciences Research Ethical Review Board (URN SR2022‐0124) of the Royal Veterinary College.

## Supporting information



Supporting Information

Supporting Information

## Data Availability

The data that support the findings of this study are available on request from the corresponding author. The data are not publicly available due to privacy or ethical restrictions.
