# Supplementary material for: Assessment of current colostrum feeding practices and passive transfer of immunity in dairy herds in Great Britain: A cross‐sectional study
Source: Vet Rec. 2025 Nov 26;199(6):e262–71. doi: 10.1002/vetr.6025 (PMC13587255; doi:10.1002/vetr.6025)
Supplement: Supplementary file 1 [file VETR-199--s001.docx]

SUPPLEMENT S1

Description of herd transfer of passive immunity (HTPI) targets and the allocation of HTPI scores:

HTPI targets were assigned based upon the benchmarks suggested by Lombard et al (2020) of:

- <10% calves with an STP <51 g/L
- <20% calves with an STP between 51-57 g/L
- >30% calves with an STP between 58-61 g/L
- >40% calves with a STP ≥62g/L

A HTPI score (0-4) was allocated for each herd based upon the number of HTPI targets that were achieved:

| HTPI Score | Definition |
| --- | --- |
| 0 | A herd did not achieve any of the suggested HTPI targets. |
| 1 | A herd achieved one of the suggested HTPI targets  For example, a HTPI score of one would be given to a herd that had <10% calves with an STP <51 g/L but did not achieve any of the other HTPI targets |
| 2 | A herd achieved two of the suggested HTPI targets  For example, a HTPI score of two would be given to a herd that had <10% calves with an STP <51 g/L and <20% calves with an STP between 51-57 g/L |
| 3 | A herd achieved three of the suggested HTPI targets  For example, a HTPI score of three would be given to a herd that had <10% calves with an STP <51 g/L and <20% calves with an STP between 51-57 g/L and >30% calves with an STP between 58-61 g/L |
| 4 | A herd achieved all four of the suggested HTPI targets |

The questionnaire provided to survey respondents enrolled prospectively

| Farm Profile |  | **Farm ID** |  |
| --- | --- | --- | --- |

Farm Name

Farm Address

Postcode

| Tel | Email |
| --- | --- |

***1. Herd Details***

| Farm type |  | Dairy |  |  | Mixed |  |  | Other: |  |
| --- | --- | --- | --- | --- | --- | --- | --- | --- | --- |

| Enterprise type |  | Conventional |  |  | Organic |  |  |
| --- | --- | --- | --- | --- | --- | --- | --- |

| Calving pattern |  | Spring |  |  | Autumn |  |  | Multi-block: |  |  | AYR: |  |
| --- | --- | --- | --- | --- | --- | --- | --- | --- | --- | --- | --- | --- |

| Predominant breed(s) |  |  |
| --- | --- | --- |

| Total number of milking cows (incl. dry cows, excl. youngstock) |  |
| --- | --- |

| Average first lactation 305-day yield (litres) |  |
| --- | --- |

| Average herd 305-day yield (litres) |  |
| --- | --- |

| Is the farm currently under bTB movement restrictions? |  | Yes |  |  | No |  |
| --- | --- | --- | --- | --- | --- | --- |

| Are the pregnant cows vaccinated with any products intended to improve calf health? |  |
| --- | --- |

| If yes, which product are the pregnant cows vaccinated with? |  |
| --- | --- |

***2. Labour details***

| Number of people involved in day-to-day care of the youngstock |  |
| --- | --- |

| What age is the primary calf rearer? |  | 21-30 |  |  | 31-40 |  |  | 41-50 |  |  | >50 |  |  | Other |  |
| --- | --- | --- | --- | --- | --- | --- | --- | --- | --- | --- | --- | --- | --- | --- | --- |
|  |  |  |  |  |  |  |  |  |  |  |  |  |  |  |  |

| Does the primary calf rearer have responsibilities on the farm other than the youngstock? |  | Yes |  |  | No |  |
| --- | --- | --- | --- | --- | --- | --- |
|  |  |  |  |  |  |  |

***3. Calving management***

| How long after birth is the calf separated from the dam? (minutes) |  |
| --- | --- |

Where do calvings mainly take place?

***4. Colostrum***

***4.1 Intake and feeding method***

How long after birth is colostrum harvested from the cow? (hours after birth)

How long after birth is colostrum fed for the first time? (hours after birth)

What volume of colostrum fed for the first time? (litres)

By what method are calves fed colostrum?

| Are calves fed a second feeding of colostrum? |  | Yes |  |  | No |  |  | Sometimes |  |
| --- | --- | --- | --- | --- | --- | --- | --- | --- | --- |
|  |  |  |  |  |  |  |  |  |  |

If sometimes, under what circumstances are calves given a second feed of colostrum?

How long after birth are calves given a second feed of colostrum? (hours)

| What quantity of colostrum is fed during the second feeding? (litres) |  |
| --- | --- |

| Do you feed transition colostrum/milk to calves? |  | Yes |  |  | No |  |  |
| --- | --- | --- | --- | --- | --- | --- | --- |

***4.2 Quality, storage and immunity transfer***

| Where do you store colostrum? |  | Colostrum isn’t stored |  |  |  |
| --- | --- | --- | --- | --- | --- |
|  |  |  |  |  |  |
|  |  | Fridge |  |  |  |
|  |  |  |  |  |  |
|  |  | Freezer |  |  |  |
|  |  |  |  |  |  |
|  |  | At room temperature |  |  |  |
|  |  |  |  |  |  |
|  |  | Other |  | | |

| Do you feed pooled colostrum? |  | Yes |  |  | No |  |  |
| --- | --- | --- | --- | --- | --- | --- | --- |

| Is the quality of colostrum tested prior to feeding |  | Yes |  |  | No |  |  | Sometimes |  |
| --- | --- | --- | --- | --- | --- | --- | --- | --- | --- |
|  |  |  |  |  |  |  |  |  |  |

If sometimes, under what circumstances is colostrum quality tested?

| What method do you use to test the quality of colostrum? |  | N/A |  |  |  |
| --- | --- | --- | --- | --- | --- |
|  |  |  |  |  |  |
|  |  | Hydrometer |  |  |  |
|  |  |  |  |  |  |
|  |  | Brix refractometer |  |  |  |
|  |  |  |  |  |  |
|  |  | Visual assessment |  |  |  |
|  |  |  |  |  |  |
|  |  | Other |  | | |

What is the minimum level quality of colostrum you will use as the first feed to a calf?

What do you do if the quality of colostrum is not sufficient?

***5. Veterinary involvement***

| Before this study, have blood samples ever been taken from calves to determine the extent of transfer of passive immunity? |  | Yes |  |  | No |  |  |
| --- | --- | --- | --- | --- | --- | --- | --- |
|  |  |  |  |  |  |  |  |

| If yes, how often are samples usually taken? |  | Less than once a year | |  |  |  |
| --- | --- | --- | --- | --- | --- | --- |
|  |  |  | |  |  |  |
|  |  | Every 6-12 months | |  |  |  |
|  |  |  | |  |  |  |
|  |  | Every 3-6 months | |  |  |  |
|  |  |  | |  |  |  |
|  |  | More than every 3 months | |  |  |  |
|  |  |  | |  |  |  |
|  |  | Other |  | | | |

How often do routine veterinary visits occur on your farm?

| How often do you receive veterinary advice regarding calf health? |  | Less than annually | | |  | |  |  |
| --- | --- | --- | --- | --- | --- | --- | --- | --- |
|  |  |  | | |  | |  |  |
|  |  | Annually | | |  | |  |  |
|  |  |  | | |  | |  |  |
|  |  | Monthly | | |  | |  |  |
|  |  |  | | |  | |  |  |
|  |  | Weekly | | |  | |  |  |
|  |  |  |  |  | |  |  |  |
|  |  | Other |  | | | | | |

| How would you rate your vets interest in the health of your calves?  *(Please circle the most accurate descriptor)* | | | | |
| --- | --- | --- | --- | --- |
| Not at all interested | Slightly interested | Moderately interested | Very interested | Extremely interested |

| How would you rate your level of agreement with the statement, “I would you like more support from my veterinarian about calf health” *(Please circle the most accurate descriptor)* | | | | |
| --- | --- | --- | --- | --- |
| Strongly disagree | Disagree | Neither agree nor disagree | Agree | Strongly agree |

Number of responses to individual questions by survey respondents and methods of categorisation

| Question | Number of responses* | Categorised? |
| --- | --- | --- |
| *Farm Type* | 33/33 | *Dairy Dairy & other* |
| *Enterprise Type* | 32/33 | *Conventional Organic* |
| *Calving Pattern* | *33*/33 | *Autumn Spring All-year-round Multi-block* |
| *Predominant Breed* | *29*/33 | *Pedigree Holstein Holstein-Friesian Crossbred* |
| *Total No. Milking cows* | *33*/33 | *-* |
| Average first lactation 305-day yield (litres) | 31/33 | *-* |
| Average herd 305-day yield (litres) | 33/33 | *-* |
| *Under bTB movement restrictions* | *33*/33 | *Yes/No* |
| *Vaccination of pregnant cows* | *33*/33 | *Yes/No* |
| *(if yes) Vaccine used* | *21/21* | *Categorised by product used* |
| *No. people involved in youngstock care* | *27*/33 | *-* |
| *Age of calf rearer* | *33*/33 | *21-30, 31-40. 41-50, >50 and other* |
| *Does the calf rearer have responsibilities other than youngstock* | *32*/33 | *Yes/no* |
| *How long after birth is the calf separated from the dam (minutes)* | *32*/33 | *Minimum and maximum* |
| *Where do calvings take place* | *Location: 30*/33  *Group or individual: 31*/33 | *Dedicated calving area: Yes/no*  *Group/individual* |
| *How long after birth is colostrum harvested from the cow? (hours after birth)* | *17*/33 | *Minimum and maximum* |
| *How long after birth is colostrum fed for the first time? (hours after birth)* | *27*/33 | *Minimum and maximum* |
| *What volume of colostrum fed for the first time? (litres)* | *25*/33 | *Minimum and maximum* |
| *By what method are calves fed colostrum?* | *32*/33 | *Left to suckle Bottle Oesophageal tube* |
| *Are calves fed a second feeding of colostrum?* | *31*/33 | *Yes/no* |
| *If sometimes, under what circumstances are calves given a second feed of colostrum?* | *6/6* | *-* |
| *How long after birth are calves given a second feed of colostrum? (hours)* | *24*/33 | *Minimum and maximum* |
| *What quantity of colostrum is fed during the second feeding? (litres)* | *24*/33 | *Minimum and maximum* |
| *Do you feed transition colostrum/milk to calves?* | *33/33* | *Yes/no* |
| *Where do you store colostrum* | *32*/33 | *Colostrum isn’t stored Fridge Freezer At room temperature Other* |
| *Do you feed pooled colostrum?* | *33*/33 | *Yes/no* |
| *Is the quality tested prior to feeding* | *33*/33 | *Yes, sometimes, no* |
| *If sometimes, under what circumstances is colostrum quality tested?* | *6/6* | *-* |
| *What method do you use to test the quality of colostrum?* | *29*/33 | *Brix Hydrometer Colostroballs Visually* |
| *What is the minimum level quality of colostrum you will use as the first feed to a calf?* | *25*/33 | *-* |
| *What do you do if the quality of colostrum is not sufficient?* | *26*/33 | *Mix with colostrum replacer Feed colostrum replacer (only) Feed stored colostrum or available colostrum from an alternative cow  (including pooled) Increase the volume fed Feed anyway* |
| *Before this study, have blood samples ever been taken from calves to determine the extent of passive transfer of immunity?* | *33*/33 | *Yes/no* |
| *If yes, how often are samples usually taken?* | *31*/33 | *More than every 3 months Every 3-6 months Every 6-12 months Less than once a year* |
| *How often do routine veterinary visits occur on your farm?* | *31*/33 | *More often than weekly Weekly Fortnightly Less often than fortnightly* |
| *How often do you receive veterinary advice regarding calf health?* | *31*/33 | *Weekly Monthly Annually* |
| How would you rate your vets interest in the health of your calves? | 31/33 | *Moderately interested Very interested Extremely interested* |
| How would you rate your level of agreement with the statement, “I would you like more support from my veterinarian about calf health” | 31/33 | Strongly disagree Disagree Neither agree nor disagree Agree |

*A total of 33 respondents completed the questionnaire.

References

1.	Lombard J, Urie N, Garry F, et al. Consensus recommendations on calf- and herd-level passive immunity in dairy calves in the United States. J Dairy Sci. 2020;103:7611-7624.
