# Supplementary material for: Assessment of current colostrum feeding practices and passive transfer of immunity in dairy herds in Great Britain: A cross‐sectional study
Source: Vet Rec. 2025 Nov 26;199(6):e262–71. doi: 10.1002/vetr.6025 (PMC13587255; doi:10.1002/vetr.6025)
Supplement: Supplementary file 2 [file VETR-199--s002.docx]

Supplement S2

TABLE S1 List of explanatory variables available for inclusion within initial univariate analysis

Variable

Vet practice

Farm type

Calving pattern

Predominant breed

Herd size

Heifer 305d milk yield

Herd 305d milk yield

bTB restrictions*

Dam vaccination

Age of the calf rearer

Calf rearer responsibilities

Minimum calf separation time

Maximum calf separation time

Calving location

Individual or group calving pen

Calving area

Minimum duration until colostrum is harvested post calving

Maximum duration until colostrum is harvested post calving

Minimum duration until colostrum is fed post calving

Maximum duration until colostrum is fed post calving

First colostrum feed volume

Colostrum feeding method

Second colostrum feed given

Minimum duration until second feed is given

Maximum duration until second feed is given

Minimum quantity fed during second feed

Maximum quantity fed during second feed

Transition milk feeding to calves

Is colostrum quality tested

Minimum quality colostrum that is fed

Alternative if colostrum is inadequate

Feeding colostrum replacer

Storing refrigerated colostrum

Storing frozen colostrum

Feeding pooled colostrum

Previously collecting blood samples for transfer of passive immunity (TPI)

Frequency of blood sampling for TPI

Frequency of routine veterinary visits

Vets interest in calf health

Agreement with the statement ““I would you like more support from my veterinarian about calf health”

^*^Statutory movement restrictions associated with M*ycobacterium bovis (bTB)* test status may be implemented under UK legislation

TABLE S2 Explanatory variables eligible for inclusion within the multivariate model and their associated chi-square values

| Variable | Chi^2^ |
| --- | --- |
| Practice | 0.075 |
| Age of the calf rearer | 0.06 |
| Calving location | 0.25 |
| Minimum duration until colostrum is fed post calving | 0.21 |
| Minimum duration until second feed is given | 0.14 |
| Maximum duration until second feed is given | 0.14 |
| Storing refrigerated colostrum | 0.25 |
| Storing frozen colostrum | <0.01 |
| Feeding pooled colostrum | 0.05 |
| Feeding colostrum replacer | <0.01 |
| Transition milk feeding to calves | 0.06 |
